# Effect of rs4719839 polymorphism on risk of ventilator‐associated pneumonia, expression of microRNA‐148 and autophagy‐related 16‐like 1 (ATG16L1)

**DOI:** 10.1111/jcmm.15824

**Published:** 2020-09-17

**Authors:** Shu‐peng Wang, Wen Li, Chen Li, Xue‐yan Duan, Jun Duan

**Affiliations:** ^1^ Surgical Intensive Care Unit China‐Japan Friendship Hospital Beijing China; ^2^ Department of Internal Medicine The University of Texas Health Science Center at Houston McGovern Medical School Houston TX USA

**Keywords:** ATG16L1, microRNA, single nucleotide polymorphism, ventilator‐associated pneumonia

## Abstract

MiR‐148 is a negative regulator of autophagy 16‐like 1 (ATG16L1), a gene implicated in the pathogenesis of ventilator‐associated pneumonia (VAP). Therefore, the role of miR‐148 polymorphism in the pathogenesis of VAP was studied here. The expression of miR‐148, ATG16L1, Beclin‐I, LC3‐II, TNF‐α and IL‐6 in serum and peripheral blood mononuclear cells (PBMCs) of VAP patients was detected to study their relationship in the pathogenesis of VAP. Chronic obstructive pulmonary disease patients carrying the AA/AG genotypes of miR‐148 rs4719839 single nucleotide polymorphism (SNP) were more prone to VAP due to the higher expression of miR‐148, TNF‐α and IL‐6 along with suppressed expression of ATG16L1, Beclin‐I and LC3‐II in their serum and PBMCs. Transfection of miR‐148 mimics to primary PBMCs genotyped as GG and AA decreased the expression of ATG16L1, Beclin‐I and LC3‐II. Finally, cells carrying the AA genotype of rs4719839 SNP were more sensitive to the role of LPS stimulation in suppressing ATG16L1, Beclin‐I and LC3‐II expression while activating TNF‐α and IL‐6 expression. Our work presented detailed evidence, suggesting that the rs4719839 polymorphism can affect the risk of VAP.

## 1 INTRODUCTION

Ventilator‐associated pneumonia (VAP) was shown to affect over 30% of ICU patients requiring mechanical ventilation.^1^ VAP can also lead to severe complications including acute respiratory distress syndrome, delirium, aspiration, venous thromboembolism, lung oedema and atelectasis, thus significantly increasing the mortality and morbidity of patients requiring mechanical ventilation.^1^

As a pathway of catabolism conserved in many species, autophagy can degrade and recycle damaged intracellular organelles via lysosomal degradation.^2^ While the level of autophagy in normal cells is low, it can be rapidly increased upon stress exposure, such as exposure to hypoxic stress and infection by viruses and bacteria.^3^, ^4^ Autophagy exerts critical effects on various intracellular processes, including apoptosis and proliferation of many types of cells, while the disorder in autophagy results in many diseases, such as autoimmune diseases and cancers.^5^, ^6^, ^7^ The induction of autophagy can also alleviate certain neurodegenerative disorders by inhibiting the level of inflammation of immune cells including dendritic cells and macrophages.^8^, ^9^, ^10^

Autophagy‐related 16‐like 1 (ATG16L1) plays critical roles in the regulation of autophagy and formation of autophagosomes by forming a complicated complex with ATG12 and ATG5.^11^ A single nucleotide polymorphism (SNP), that is rs2241880 Thr300 Ala c.898A>G, located in ATG16L1 impairs autophagy functions to increase the risk of tuberculosis.^12^ Furthermore, NOD2 and NOD1 receptors can recruit ATG16L1 proteins upon bacterial infection to activate autophagy, while the A allele of rs2241880 impairs the activation of autophagy to increase the risk of septic shock in VAP patients.^13^

As a type of short RNAs containing about 22 nucleotides, microRNA (miRNA) possesses dual tumour‐suppressing and oncogenic properties based on actual cellular context.^14^ Deregulated expression of miRNAs can lead to multiple malignancies such as lung and breast cancer.^15^, ^16^, ^17^ The G allele of rs4719839 SNP located in miR‐148a affects the size and TNM score of certain tumours.^18^ The G allele in the rs2241880 SNP of ATG16L1 affects the activation of autophagy by modifying the polarity of ATG16L1 protein, thus increasing the risks of Crohn's disease (CD).^19^, ^20^, ^21^ Furthermore, rs2241880 SNP was identified as a risk factor in certain European population.^22^, ^23^, ^24^

It has been reported that rs2241880 polymorphism may affect the expression level of ATG16L1, while the rs4719839 polymorphism may affect the expression of miR‐148, a negative regulator of ATG16L1.^18^, ^25^ ATG16L1 has been implicated in the pathogenesis of VAP.^26^ In this study, we enrolled chronic obstructive pulmonary disease (COPD) patients with or without VAP to study the role of rs2241880 and rs4719839 polymorphisms in the pathogenesis of VAP.

## 2 MATERIALS AND METHODS

### 2.1 Human sample collection

A total of 280 patients provided consent to take part in our research, which included 142 COPD patients suffering from VAP and 132 COPD patients free of VAP who were enrolled during May 2013 to August 2016 at China‐Japan Friendship Hospital. Peripheral blood samples were collected from above patients. Information of all participants including their age, gender, APACHE II score^27^ and underlying diseases (type 2 diabetes, non‐Hodgkin's lymphoma, Parkinson's disease, coronary heart disease, heart failure and chronic kidney diseases) was collected and summarized. Institutional ethical committee has approved the protocol of this study.

### 2.2 Genotyping

The peripheral blood samples collected from the COPD patients with or without VAP were subjected to isolation of genomic DNA utilizing a Genomic DNA Isolation Assay Kit (Qiagen). Then, 25 ng of isolated genomic DNA from each patient was mixed with an appropriate amount of Maxima Hot Start Green Master Mix (Thermo Fisher Scientific) and corresponding primers were designed for miR‐148 rs4719839 SNP and ATG16L1 rs2241880 SNP. In the next step, real‐time PCRs were carried out on a Bio‐Rad real‐time PCR machine (Bio‐Rad) using a TaqMan Assay Kit (ABI) in accordance with the instructions provided on kit manual to determine the genotypes of miR‐148 rs4719839 SNP and ATG16L1 rs2241880 SNP in various peripheral blood samples.

### 2.3 RNA isolation and real‐time PCR

Peripheral blood samples collected from the COPD patients with or without VAP were treated to separate monocytes. Then, total RNA content in the monocytes was isolated utilizing a TRIzol reagent (Invitrogen) following the instructions of the manufacturer. In the next step, the extracted RNA was reversely transcribed to cDNA using a reverse transcription assay kit (Thermo Fisher Scientific) following kit instruction and then loaded onto the Bio‐Rad real‐time PCR machine for real‐time PCRs. The relative expression of miR‐148 (Forward primer sequence: GTTCTGAGACACTCCGA; Reverse primer sequence: GAACATGTCTGCGTATCTC), ATG16L1 (Forward primer sequence: CTACGGAAGAGAACCAGGAGCT; Reverse primer sequence: CTGGTAGAGGTTCCTTTGCTGC), TNF‐α (Forward primer sequence: CTCTTCTGCCTGCTGCACTTTG; Reverse primer sequence: ATGGGCTACAGGCTTGTCACTC) and IL‐6 (Forward primer sequence: AGACAGCCACTCACCTCTTCAG; Reverse primer sequence: TTCTGCCAGTGCCTCTTTGCTG) in each sample was calculated using the 2^‐ΔΔCt^ method in conjunction with the software equipped with the real‐time PCR machine following reaction conditions of 95°C for 15 minutes (initial activation), 94°C for 15 seconds (denaturation), 55°C for 30 seconds (followed by annealing) and 72°C for 60 seconds (final extension). The internal controls used were 5S (Forward primer sequence: CTCGCTTCGGCAGCACAT; Reverse primer sequence: TTTGCGTGTCATCCTTGCG) and GAPDH (Forward primer sequence: GTCTCCTCTGACTTCAACAGCG; Reverse primer sequence: ACCACCCTGTTGCTGTAGCCAA).

### 2.4 Cell culture and treatment

Primary PBMCs genotyped as GG and AA were obtained and cultured in DMEM containing appropriate antibiotics and 10% serum. The routine culture operation was carried out in a 5% CO_2_ incubator at 37°C and saturated humidity. Upon 80% confluency, primary PBMCs genotyped as GG and AA were divided into the following three groups: (a) NC; (b) miR‐148 mimics; and (c) miR‐148 inhibitors. The cells in the NC group were cultured in normal DMEM. The cells in the miR‐148 mimics or miR‐148 inhibitors group were transfected with 25 nM miR‐148 mimics and miR‐148 inhibitors, respectively. All transfection was carried out using Lipofectamine 2000 (Invitrogen, Carlsbad, CA) following the instructions of the manufacturer, and the transfected cells were harvested at 48 hours after the start of the transfection. In addition, to study the role of miR‐148 rs4719839 SNP in the expression of miR‐148, primary PBMCs genotyped as GG and AA were divided into a GG group and an AA group. The cells in the GG group carried the GG genotype of miR‐148 rs4719839 SNP, while the cells in the AA group carried the AA genotype of miR‐148 rs4719839 SNP. Then, the primary PBMCs genotyped as GG and AA in both groups were stimulated with LPS for 24 hours before the levels of miR‐148 expression in both groups were measured by real‐time PCR.

### 2.5 Luciferase assay

The TargetScan database was used to obtain the wild‐type sequence for the 3′‐untranslated region (UTR) of ATG16L1. Then, the mutant type or wild‐type fragments of ATG16L1 3′‐UTR containing the miR‐148 binding site were subcloned into pcDNA3.1 luciferase vectors (Promega), which were transfected into primary PBMCs genotyped as GG and AA in conjunction with miR‐148 using Lipofectamine 2000. After 48 hours of incubation, the luciferase activity of cell lysates in each well of the tissue culture plate was measured using a luciferase reporter assay (Promega) following a routine procedure recommended by the producer. The luciferase activity of the cells transfected with the mutant type or wild‐type fragments of ATG16L1 3′‐UTR was compared to determine the regulatory relationship between ATG16L1 and miR‐148 expression.

### 2.6 Western blotting analysis

The peripheral blood samples collected from the COPD patients with or without VAP, as well as the transfected cell samples, were lysed to isolate their protein content. Then, 50 µg of protein from each sample was resolved by 12% SDS‐PAGE (Invitrogen) and blotted onto appropriate polyvinylidene fluoride (PVDF) membranes (GE Healthcare). After blocking in 5% skim milk and PBS washing, the membranes were treated overnight at 4°C with antibodies against ATG16L1, Beclin‐I, LC3‐II, TNF‐α and IL‐6, respectively. Following another PBS wash, the membranes were further treated for 2 hours at 22°C with HRP‐linked secondary antibodies. All antibodies were purchased from Cell Signaling Technology. Finally, the protein bands of ATG16L1 (1:1000, # 8089S), Beclin‐I (1:1000, # 3738S), LC3‐II (1:1000, # 2775S) and IL‐6 (1:1000, # 12912S) were visualized using a Clarity ECL reagent (Bio‐Rad, Hercules, CA) to determine the relative expression of ATG16L1, Beclin‐I, LC3‐II and IL‐6 proteins.

### 2.7 ELISA

Peripheral blood samples collected from the COPD patients with or without VAP were treated to separate monocytes. Then, the expression levels of TNF‐α and IL‐6 in both the peripheral blood samples and the separated monocytes were determined using commercially available ELISA kits (Thermo Fisher Scientific) following the routine assay methods recommended in the kit instructions.

### 2.8 Statistical analysis

The regulatory relationship between ATG16L1 and miR‐148 expression was evaluated utilizing the Mann‐Whitney *U* tests. The comparisons among multiple groups were carried out using one‐way ANOVA or Student's *t* tests. The correlation between miR‐148 rs4719839 SNP and the expression level of miR‐148 as well as the correlation between ATG16L1 rs2241880 SNP and the expression level of ATG16L1 was analysed utilizing Spearman's rank tests. Multivariate logistic regression analysis was used to evaluate the association between the risk of VAP in COPD patients and the miR‐148 rs4719839 or ATG16L1 rs2241880 polymorphisms. All statistical analyses were carried out using GraphPad Prism 7.0 (GraphPad) and SPSS 21.0 (SPSS, IBM). The level of statistical significance was set to 0.05.

## 3 RESULTS

### 3.1 Patient characteristics in experimental and control groups

As shown in Table 1, information including their age, gender, APACHE II score and underlying diseases (type 2 diabetes, non‐Hodgkin's lymphoma, Parkinson's disease, coronary heart disease, heart failure and chronic kidney diseases) was collected from the COPD participants with or without VAP. Student's *t* test was carried out to compare the above characteristics between COPD + VAP and COPD‐VAP groups, and no difference was observed.

**Table 1:** Basic information of the participants of this study

Characteristics	Patients with VAP (N = 142)	Control (N = 138)	*P* value
Sex (male/female)	104/38	101/37	.942
Age (mean ± SD)	64.5 ± 15.1	66.4 ± 15.2	.883
APACHE II score (mean ± SD)	18.4 ± 5.3	18.7 ± 5.6	.793
Underlying disease (number, %)			.763
Diabetes mellitus type 2	42 (29.6)	38 (27.6)	
Coronary heart disease	30 (21.2)	26 (18.8)	
Hypertension	39 (27.6)	42 (30.3)	
Chronic kidney disease	3 (2.0)	2 (1.4)	

Abbreviations: VAP, ventilator‐associated pneumonia.

### 3.2 Rs4719839 and rs2241880 polymorphisms were not associated with the risk of VAP

Multivariate logistic regression analysis was used to evaluate the association between the risk of VAP in COPD patients and the miR‐148 rs4719839 or ATG16L1 rs2241880 polymorphisms, as shown in Table 2. Both polymorphisms showed no obvious association with the risk of VAP in COPD patients.

**Table 2:** Comparison of the alleles and genotypes of the two polymorphisms between the VAP and control groups

SNP	Genotype	Patients with VAP (N = 142)	Control (N = 138)	*P* value
‐376 G/A	GG	138 (97.2)	135 (97.8)	
	GA	4 (2.8)	3 (2.2)	
	AA	0 (0)	0 (0)	
	G allele	280 (98.6)	273 (18.9)	.742
	A allele	4 (1.4)	3 (1.1)	
‐308 G/A	GG	118 (83.1)	113 (81.9)	
	GA	22 (15.5)	23 (16.7)	
	AA	2 (1.4)	2 (1.4)	
	G allele	258 (90.8)	249 (90.2)	.653
	A allele	26 (9.2)	27 (9.8)	

Abbreviations: SNP, single nucleotide polymorphism; VAP, ventilator‐associated pneumonia.

### 3.3 AA and AG genotypes of miR‐148 rs4719839 SNP reduced the incidence of VAP in COPD patients

**Figure 1:**
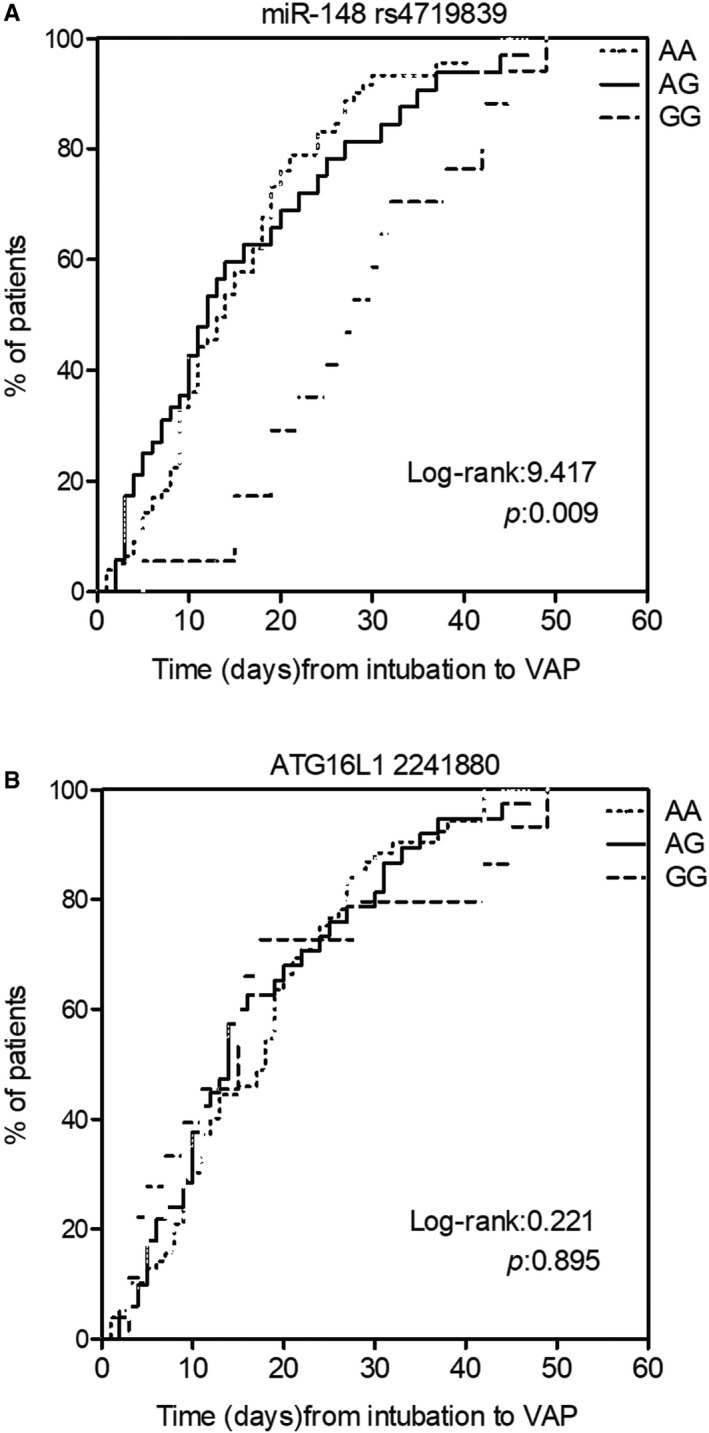
AA/AG genotypes of rs4719839 increased the risk of VAP in COPD patients. A, Higher risk of VAP in COPD patients with AA/AG genotypes of rs4719839. B, No difference in the VAP incidence in COPD patients with different genotypes of rs2241880. VAP, ventilator‐associated pneumonia

We collected 142 COPD patients suffering from VAP to evaluate the relationship between the genotypes of rs4719839/rs2241880 SNPs and the incidence of VAP. Rs4719839 AA and AG SNPs showed a higher incidence of VAP than the GG genotype (Figure 1A), while no obvious difference was found among rs2241880 AA, AG and GG genotypes (Figure 1B).

### 3.4 MiR‐148 rs4719839 AA and AG increased the expression of miR‐148, TNF‐α and IL‐6 in serum and PBMC

**Figure 2:**
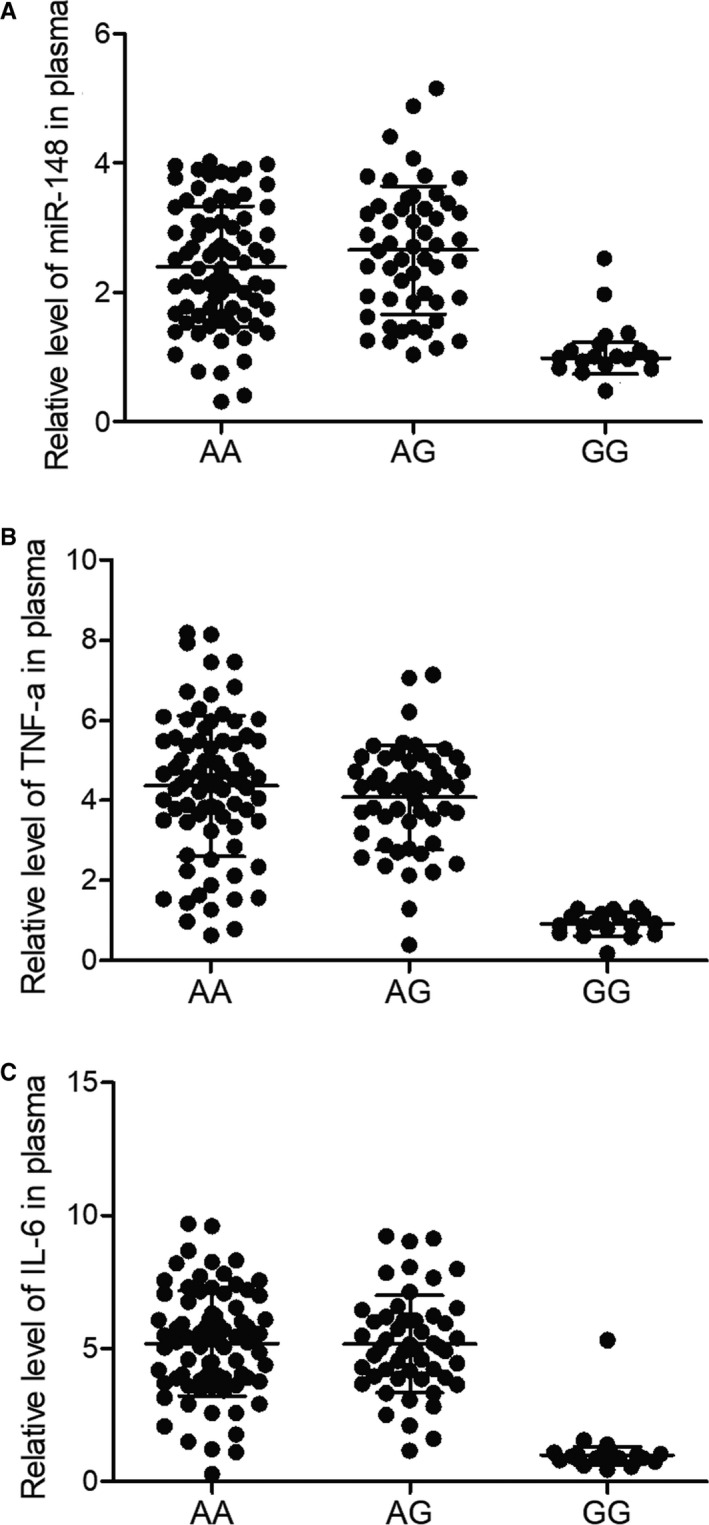
Elevated expression of miR‐148, TNF‐α and IL‐6 in the serum of VAP patients with AA/AG genotypes. A, MiR‐148 expression was increased in the serum of VAP patients with AA/AG genotypes. B, TNF‐α expression was increased in the serum of VAP patients with AA/AG genotypes. C, IL‐6 expression was increased in the serum of VAP patients with AA/AG genotypes. VAP, ventilator‐associated pneumonia

**Figure 3:**
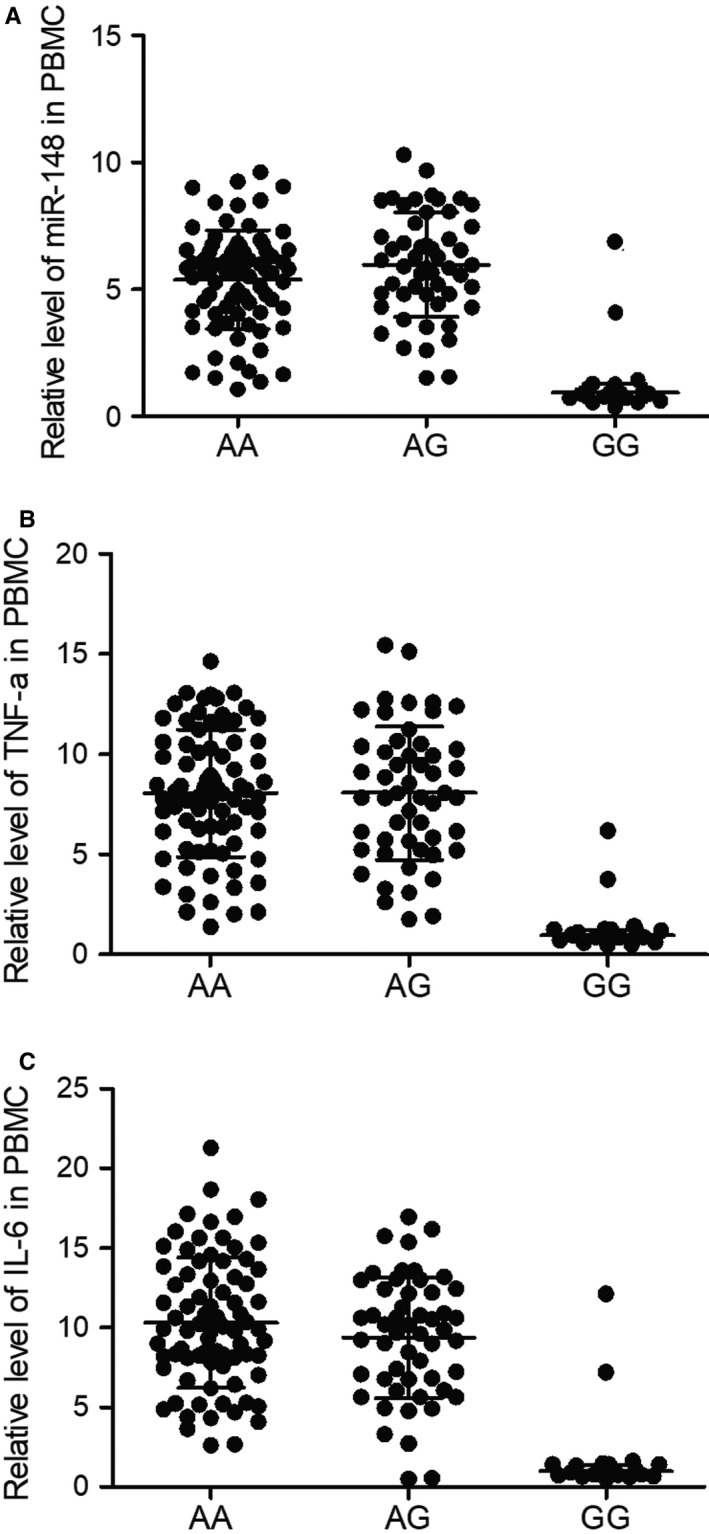
Elevated expression of miR‐148, TNF‐α and IL‐6 in the PBMCs of VAP patients with AA/AG genotypes. A, MiR‐148 expression was increased in the PBMCs of VAP patients with AA/AG genotypes. B, TNF‐α expression was increased in the PBMCs of VAP patients with AA/AG genotypes. C, IL‐6 expression was increased in the PBMCs of VAP patients with AA/AG genotypes. PBMCs, peripheral blood mononuclear cells; VAP, ventilator‐associated pneumonia

Quantitative real‐time PCR was performed to compare the expression of miR‐148 in peripheral blood samples of VAP patients carrying different rs4719839 genotypes. Increased expression of miR‐148 was observed in AA and AG groups (Figure 2A). ELISAs showed higher expression of TNF‐α (Figure 2B) and IL‐6 (Figure 2C) in peripheral blood collected from patients genotyped as AA and AG for their rs4719839 SNP. Furthermore, the up‐regulation of miR‐148, TNF‐α and IL was more pronounced in PBMC of VAP patients carrying rs4719839 AA and AG genotypes (Figure 3).

### 3.5 Differential expression of ATG16L1 in different groups

**Figure 4:**
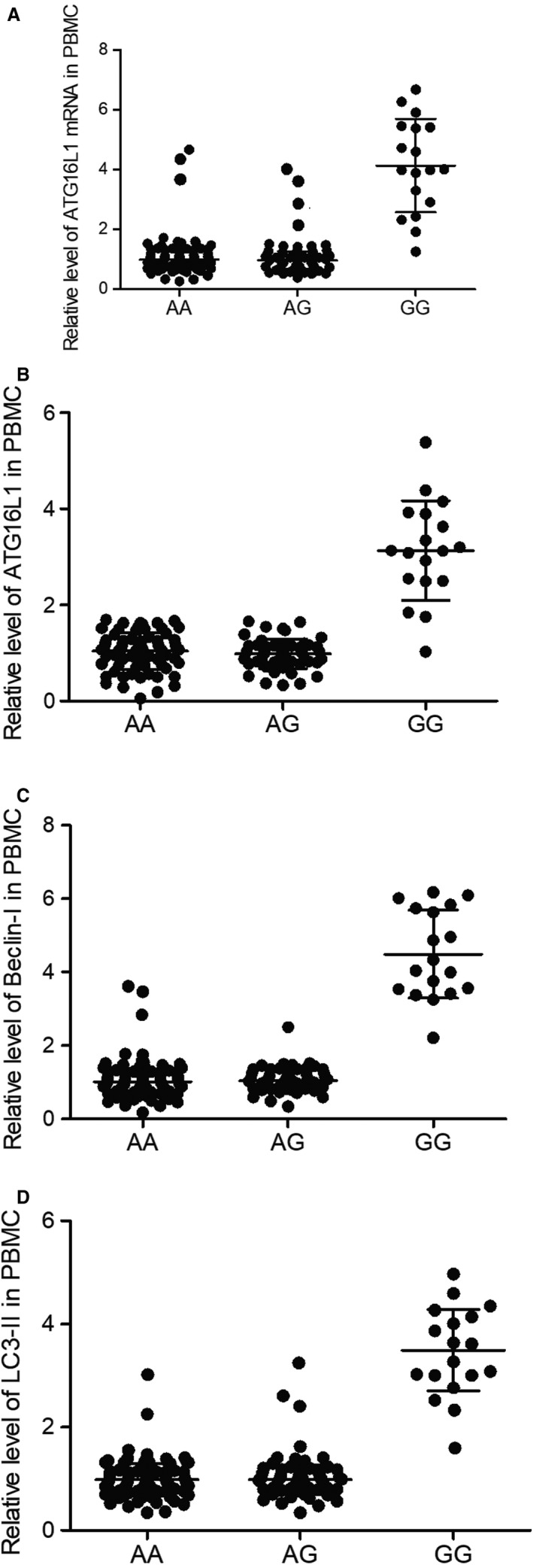
Suppressed expression of ATG16L1, Beclin‐I and LC3‐II in the PBMCs of VAP patients with AA/AG genotypes. A, ATG16L1 expression was inhibited in the PBMCs of VAP patients with AA/AG genotypes. B, ATG16L1 protein was reduced in the PBMCs of VAP patients with AA/AG genotypes. C, Beclin‐I protein was reduced in the PBMCs of VAP patients with AA/AG genotypes. D, LC3‐II protein was reduced in the PBMCs of VAP patients with AA/AG genotypes. PBMCs, peripheral blood mononuclear cells; VAP, ventilator‐associated pneumonia

Next, we evaluated the expression of ATG16L1, which has been implicated in the pathogenesis of VAP, in the PBMC of VAP patients carrying different genotypes of rs4719839 SNP. As shown in Figure 4, the mRNA (Figure 4A) and protein (Figure 4B) expression of ATG16L1 was significantly enhanced in patients with the GG genotype. Moreover, the expression of Beclin‐I (Figure 4C) and LC3‐II (Figure 4D) was also elevated in patients with the GG genotype, demonstrating a higher risk of VAP in patients with the GG genotype.

### 3.6 MiR‐148 down‐regulated ATG16L1 and other genes related to VAP

**Figure 5:**
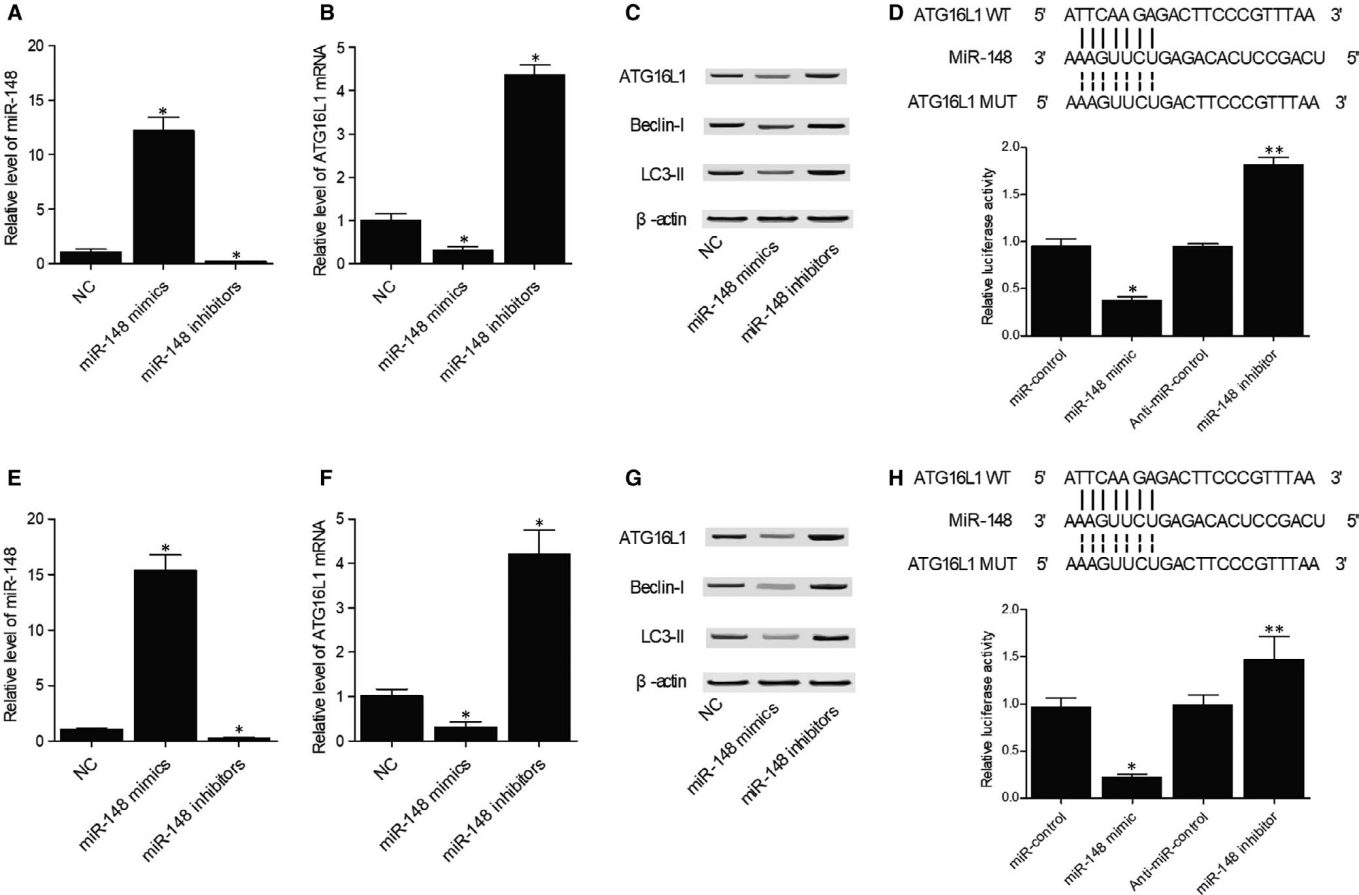
MiR‐148 down‐regulated the expression of ATG16L1 and genes related to VAP. A, MiR‐148 was overexpressed in primary PBMCs genotyped as GG transfected with miR‐148 mimics and inhibited in primary PBMCs genotyped as GG transfected with miR‐148 inhibitors (*P value < 0.05 compared with NC). B, ATG16L1 mRNA was inhibited in primary PBMCs genotyped as GG transfected with miR‐148 mimics and up‐regulated by miR‐148 inhibitors (*P value < 0.05 compared with NC). C, ATG16L1, Beclin‐I and LC3‐II proteins were down‐regulated in primary PBMCs genotyped as GG transfected with miR‐148 mimics and were up‐regulated by miR‐148 inhibitors. D, MiR‐148 mimics inhibited the luciferase activity of ATG16L1 3′ UTR, while miR‐148 inhibitors increased the luciferase activity of ATG16L1 3′ UTR in primary PBMCs genotyped as GG (*P value < 0.05 compared with miR‐control; **P value < 0.05 compared with anti‐miR‐control). E, MiR‐148 was overexpressed in primary PBMCs genotyped as AA transfected with miR‐148 mimics and inhibited by miR‐148 inhibitors (*P value < 0.05 compared with NC). F, ATG16L1 mRNA was inhibited in primary PBMCs genotyped as AA transfected with miR‐148 mimics and up‐regulated by miR‐148 inhibitors (*P value < 0.05 compared with NC). G, ATG16L1, Beclin‐I and LC3‐II proteins were down‐regulated in primary PBMCs genotyped as AA transfected with miR‐148 mimics and were up‐regulated by miR‐148 inhibitors. H, MiR‐148 mimics inhibited the luciferase activity of ATG16L1 3′ UTR, while miR‐148 inhibitors increased the luciferase activity of ATG16L1 3′ UTR in primary PBMCs genotyped as AA (*P value < 0.05 compared with miR‐control; **P value < 0.05 compared with anti‐miR‐control). PBMCs, peripheral blood mononuclear cells; VAP, ventilator‐associated pneumonia

To understand the regulatory role of miR‐148 in VAP, we transfected primary PBMCs genotyped as GG with miR‐148 mimics and inhibitors (Figure 5A). ATG16L1 mRNA was remarkably decreased in primary PBMCs genotyped as GG transfected with miR‐148 mimics and notably elevated in primary PBMCs genotyped as GG transfected with miR‐148 inhibitors (Figure 5B). Accordingly, the expression of ATG16L1, Beclin‐I and LC3‐II proteins was also reduced in primary PBMCs genotyped as GG transfected with miR‐148 mimics and enhanced in primary PBMCs genotyped as GG transfected with miR‐148 inhibitors (Figure 5C). Analysis of miR‐148 and ATG16L1 sequences found a potential binding site of miR‐148 on the 3′ UTR of the ATG16L1. The results of luciferase assays revealed the inhibitory effect of miR‐148 on ATG16L1 expression by binding to the 3′ UTR of ATG16L1 (Figure 5D). We also performed above assays in primary PBMCs genotyped as AA and obtained similar results (Figure 5E‐H).

### 3.7 Divergent response of cells carrying different genotypes of rs4719839 SNP upon LPS stimulation

**Figure 6:**
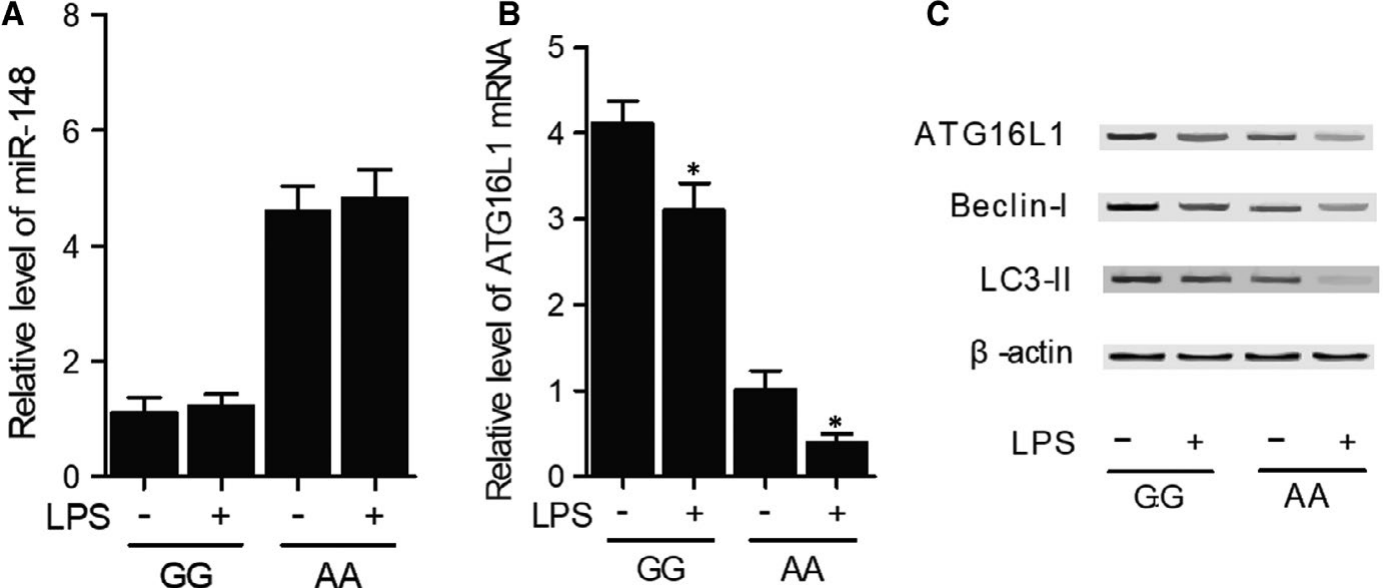
Divergent response of cells carrying different genotypes of rs4719839 upon LPS stimulation. A, No obvious difference in expression was detected after both primary PBMCs genotyped as GG and AA were stimulated by LPS. B, Decrease in ATG16L1 mRNA expression was more pronounced after LPS stimulation in primary PBMCs genotyped as AA carrying AA genotype of rs4719839 (*P value < 0.05 compared with unstimulated PBMCs). C, Down‐regulation of ATG16L1, Beclin‐I and LC3‐II protein was more pronounced after LPS stimulation in primary PBMCs genotyped as AA carrying AA genotype of rs4719839. PBMCs, peripheral blood mononuclear cells

Primary PBMCs genotyped as GG and AA carrying GG and AA genotypes of rs4719839 SNP were subjected to LPS stimulation to compare their responses. MiR‐148 expression was significantly higher in primary PBMCs genotyped as AA carrying AA genotype, but no obvious change was detected when the cells were treated with LPS (Figure 6A). ATG16L1 expression was elevated in primary PBMCs genotyped as GG carrying GG genotype and was decreased after LPS was added to the cells. In addition, the decrease in ATG16L1 expression was more pronounced in the primary PBMCs genotyped as AA carrying AA genotype (Figure 6B). Besides, the expression of ATG16L1, Beclin‐I and LC3‐II proteins showed the same trend as that of ATG16L1 mRNA in different groups (Figure 6C).

## 4 DISCUSSION

In this study, we enrolled a group of COPD patients carrying differential genotypes of rs4719839 (AA/AG/GG) and rs2241880 (AA/AG/GG) SNPs to evaluate their role in the risk of VAP. Different genotypes of rs4719839 SNP but not rs2241880 SNP showed strong correlation with the incidence of VAP. Additionally, we collected serum and monocytes from the peripheral blood of VAP patients carrying different genotypes of rs4719839 (AA/AG/GG) to check their expression of miR‐148, TNF‐α and IL‐6. Significantly higher expression of miR‐148, TNF‐α and IL‐6 was observed in patients carrying AA and AG genotypes. The rs4719839 SNP positioned in the promoter of miR‐148a affects the binding between miR‐148a and several transcription factors by affecting the post‐transcriptional regulation of miR‐148a, thus affecting tumour metastasis and growth.^18^ The G allele of rs4719839 SNP can also affect the TNM scores and size of several tumours.^18^ In this study, we assessed the expression of ATG16L1, Beclin‐I and LC3‐II in PBMCs collected from VAP patients carrying different genotypes of rs4719839 (AA/AG/GG). Elevated expression of ATG16L1, Beclin‐I and LC3‐II was detected in VAP patients carrying GG genotype.

The Thr300Ala polymorphism located in ATG16L1 increases the risk of CD by substantially increasing Atg16L1 cleavage and reducing the protein expression of Atg16L1.^19^, ^28^, ^29^ Patients and mice carrying the homozygous G alleles of rs2241880 SNP of ATG16L1 also showed increased risks of goblet cell disorders.^30^, ^31^ Moreover, intracellular proteins NOD2 and NOD1 exert critical effects on autophagy induction upon bacterial invasion by recruiting ATG16L1 proteins to the site of bacterial infection on cell membrane.^31^ In addition, the A allele of rs2241880 SNP of ATG16L1 can increase the risks of septic shock in VAP patients.^32^ In this study, we stimulated primary PBMCs genotyped as GG and AA with LPS. No difference in miR‐148 expression was found in both cells before and after LPS stimulation, but LPS stimulation decreased expression of ATG16L1, Beclin‐I and LC3‐II while increasing TNF‐α and IL‐6 expression in both cells. However, the changes of gene expression after LPS stimulation were more pronounced in primary PBMCs genotyped as AA carrying the AA genotype compared with primary PBMCs genotyped as GG carrying GG genotype.

MiR‐148 is involved in the infection of V. harveyi by targeting MyD88 in many fish species to reduce the level of inflammation.^33^ MiR‐148 overexpression also substantially attenuates the synthesis of inflammatory cytokine via targeting MyD88 functions directly to inhibit NF‐κB signalling.^33^ As a miR‐148a target, Gas1 promotes Hh signalling activation to reduce the activity of autophagy in HSC. Upon starvation of cells, miR‐148a can also induce autophagy by suppressing the level of Hh signalling, while the overexpression of miR‐148a inhibits the proliferation of HSC and promotes their apoptosis.^34^ In this study, we transfected primary PBMCs genotyped as GG and AA with miR‐148 mimics and inhibitors to check their effects on the expression of ATG16L1, Beclin‐I and LC3‐II. miR‐148 mimics remarkably decreased the expression of ATG16L1, Beclin‐I and LC3‐II, while miR‐148 inhibitors notably up‐regulated the expression of ATG16L1, Beclin‐I and LC3‐II. Luciferase assays demonstrated that miR‐148 inhibited ATG16L1 expression by binding to its 3′ UTR. In a past study, liposome sedimentation experiments showed that the binding affinity between lipid and ATG16L1 is weak, indicating that other factors including post‐translational ATG16L1 modifications are involved to promote the recruitment of ATG16L1 on cell membrane.^35^ Therefore, the formation of autophagosomes depends on ATG16L1 displacement and the activation of its upstream pathways. Moreover, the elongation of autophagosomes and their curvature can promote their release.^35^ For example, the activation of the ATG16L1 signalling can protect cells against ER‐induced stress and reduce IKK/NF‐κB‐induced pro‐inflammatory events.^36^ The characterization studies on IRGM and ATG16L1 mutants also pointed to the essential role of autophagosomes in controlling gastrointestinal inflammation and the progression of CD. Moreover, the inhibited ATG16L1 expression can increase the in vivo pro‐inflammatory responses upon NOD2 activation.^37^ Other studies also showed that ATG16L1 deletions in mice impaired the autophagy machinery and increased the severity of inflammation in CD.^30^, ^38^

## 5 CONCLUSION

Taken together, our study demonstrates that the rs4719839 SNP in ATG16L1 can influence the expression of miR‐148 and negatively regulate ATG16L1 expression to increase the risk of VAP.

## CONFLICT OF INTEREST

None.

## AUTHOR CONTRIBUTIONS

**Shu‐peng Wang:** Conceptualization (equal); Investigation (equal); Methodology (equal); Writing‐original draft (equal). **Wen Li:** Formal analysis (equal); Investigation (equal); Methodology (equal); Software (equal); Writing‐original draft (equal). **Chen Li:** Formal analysis (equal); Investigation (equal); Visualization (equal). **Xue‐yan Duan:** Methodology (equal); Software (equal); Writing‐review & editing (equal). **Jun Duan:** Conceptualization (equal); Funding acquisition (equal); Project administration (equal); Resources (equal); Supervision (equal); Writing‐original draft (supporting); Writing‐review & editing (equal).

## Data Availability

The data that support the findings of this study are available from the corresponding author upon reasonable request.
